# Proton Pump Inhibitors Interfere With Zinc Absorption and Zinc Body Stores

**DOI:** 10.4021/gr379w

**Published:** 2011-11-20

**Authors:** Christopher P. Farrell, Melissa Morgan, David S. Rudolph, Austin Hwang, Nicole E. Albert, Mary C. Valenzano, Xuexuan Wang, Giancarlo Mercogliano, James M. Mullin

**Affiliations:** a Department of Gastrointestinal Medicine, Lankenau Medical Center, 100 Lancaster Avenue, Wynnewood, PA 19096, USA; b Department of Internal Medicine, Lankenau Medical Center, 100 Lancaster Avenue, Wynnewood, PA 19096, USA; c Lankenau Institute for Medical Research, 100 Lancaster Avenue, Wynnewood, PA 19096, USA

**Keywords:** Acid, Stomach, Intestine, pH, Proton pump inhibitor, Zinc, Trace metal, Nutrition, Colic

## Abstract

**Background:**

Proton pump inhibitors (PPIs) cause a sharp elevation of gastro-duodenal luminal pH which in turn has resulted in reports of reduced absorption of magnesium and certain other nutrients.

**Methods:**

Gastroesophageal reflux disease (GERD) patients on long-term PPI therapy (> 6 months) or healthy test subjects (not on any acid preventive or neutralizing medication) were administered oral doses of zinc gluconate (26.2 mg zinc, twice daily) for 14 days followed by 5 cc venous blood samples. Plasma was analyzed for total zinc content by atomic absorption spectrophotometry. Baseline plasma and red blood cell zinc levels were also measured in these two groups when not taking any zinc supplementation.

**Results:**

Plasma zinc levels of healthy controls increased by 126% during the period of zinc supplementation compared to only a 37% increase for individuals on long-term PPI therapy. On their normal diet (with no zinc supplementation), PPI-users had a 28% lower plasma zinc level than healthy controls (P < 0.005).

**Conclusions:**

PPI use dramatically reduces supplemental zinc uptake and can result in decreased zinc body stores. Certain individuals on long-term PPI therapy, such as infants being treated for colic, may be at risk for decreased systemic levels of trace metals needed for developmental, regenerative and immunological requirements.

## Introduction

In 2009, 110 million prescriptions were written for proton pump inhibitors (PPIs) in the United States, making them the third largest class of drug prescribed in the country [1]. PPIs have long been regarded as safe and efficacious [2]. However, with recent literature suggesting PPIs may interfere with the absorption of certain nutrients through the small bowel, as well as potentially predispose one to certain infections, the long-term safety of PPIs has become a concern [2, 3].

By irreversibly deactivating the hydrogen-potassium adenosine triphosphatase present on the luminal aspect of parietal cell membranes, PPIs raise the gastroduodenal luminal pH from approximately 1.5 to 6.0 [2, 4]. Among other effects, this increase in pH is thought to interfere with the absorption of certain trace metals, micronutrients, and vitamins [2]. There are several conflicting reports regarding the connection between chronic PPI use and calcium malabsorption contributing to hip fractures [5]. The large nested case-control study by Yang and colleagues revealed a significantly increased risk of hip fracture in patients chronically on PPIs [6]. However, in a study of more than 1 million patients performed by the Women’s Health Initiative, no association between PPI use and hip fractures was found, with only a modest association with spine, arm, and wrist fractures identified [7].

Micronutrient deficiencies, such as B12 and iron, have been described as a result of the hypochlorhydria seen with chronic PPI use [2]. Gastric acid participates in the dissociation of iron from food and further reduces it to a more soluble form, aiding in its uptake by the intestinal mucosa [3]. Acid is also required for the release of vitamin B12 from ingested proteins to allow for absorption [3, 8]. There is evidence that PPI use may also lower vitamin C levels, by decreasing its bioavailability as a free acid after ingestion [3].

There is a belief that if the stomach lumen loses its protective acidity, ingested pathogenic organisms can better survive and proliferate [2]. A Dutch study in 2004 looked at 364,683 patients and showed a 4.5 times higher risk of community acquired pneumonia in patients on PPIs or histamine-2 (H2) receptor antagonists [9]. In a meta-analysis of 12 papers in 2007, there was an increased risk of Clostridium difficile; as well as Campylobacter, Salmonella, and other enteric infections in patients on acid-suppressive medications [10]. The mechanism by which PPIs may predispose patients to Clostridium difficile is thought to occur by allowing the spore to convert to its vegetative form, and thereby survive intraluminally. However, effects of PPI-induced micronutrient deficiencies on immune system function should also be considered.

The hypochlorhydria and elevated pH that has been shown to decrease calcium and magnesium absorption by the duodenum could have the same effect on other divalent cations, such as zinc. It has been reported that zinc is readily absorbed at a low pH, but not as easily at elevated pHs [11]. Zinc plays an essential role in physiologic and immunologic processes [12]. Deficiencies of this micronutrient can lead to growth retardation in children and adolescents, hypogonadism in men, skin changes, poor appetite, delayed wound healing, taste abnormalities, mental lethargy, and anergy [13]. Conversely, the most worrisome complication of zinc excess is copper depletion, which can lead to anemia, nerve damage, and bone loss [14].

PPI-mediated zinc deficiency in body stores has not been reported or investigated in the literature. In our three part study, the primary endpoints were to show that long-term daily PPI use interferes with the absorption of zinc supplementation, as well as causing a clinically and statistically significant decrease in zinc body stores.

## Methods

### Study design

These studies were conducted at the Lankenau Institute for Medical Research and the Lankenau Hospital in Wynnewood, PA. The protocol was approved by the Lankenau Medical Center Institutional Review Board and all test subjects were required to sign an informed consent at the time of enrollment.

### Test subjects

The aim of the first portion of the study was to see if zinc supplementation would lead to an increase in zinc plasma levels, while having a minimal effect on serum copper. Fifteen healthy controls (all greater than 17 years of age) were recruited without regard to gender or ethnicity. Exclusion criteria included any prior or current history of a serious gastrointestinal disease, malabsorptive disease, or surgery involving the small or large bowel; current or recent use of glucocorticoids or immunosuppressant’s; current use of antibiotics, hormone replacement therapy, amiloride or cholestyramine; current pregnancy or nursing; any illness within the past 14 days; any clotting disorder or current use of anticoagulation; any mineral or vitamin use within 3 days of enrollment; renal disease; and diabetes. Patients with gastroesophageal reflux disease (GERD) and those on PPIs were not excluded from this study.

The second portion of the trial compared the efficacy of zinc supplementation among controls and individuals on long-term PPI therapy. Five healthy controls and five subjects who have been on a PPI for at least 6 months > 17 years of age were recruited without regard to gender, ethnicity, or PPI type. The control subjects had no use of PPIs, H2 antagonists, or antacids for at least one month prior to the study. Exclusion criteria remained the same as above. The subjects on chronic PPIs possessed no other GI disease than GERD.

The third portion of the study examined the implications of chronic PPI use on baseline plasma zinc levels. Ten healthy controls and ten subjects taking PPI medication for at least six months (all greater than 17 years of age) were recruited without regard to gender, ethnicity, or PPI type. The control subjects again had no use of acid-suppressing medication for at least one month prior to the study. Exclusion criteria remained the same as for the previous studies and GERD was the only gastrointestinal disease among the chronic PPI users. All subjects were required to have a daily diet containing at least one serving of meat or dairy and one serving of vegetables, due to the high zinc content of meat and the low zinc content of typical vegetarian diets.

### Materials

Zinc gluconate lozenges (Cold-Eeze) were donated by Prophase Labs (Doylestown, PA, USA). Each lozenge contains a zinc equivalent of 13.3 mg. Plasma zinc and serum copper levels were measured by Quest Diagnostics (Chantilly, VA, USA) with the assistance of Main Line Health Laboratories (Wynnewood, PA, USA). Zinc levels in red blood cells were determined by means of atomic absorption spectroscopy (AAS) of supernatants of lysed red blood cells at St. Joseph’s University’s Department of Chemistry (Philadelphia, PA, USA).

### Measurement of zinc levels

Following a screening medical history questionnaire, fifteen healthy controls who met inclusion criteria for the initial portion of the study, provided four 5 mL samples of fasting venous blood. These were sent to Quest Diagnostics for analysis of baseline plasma zinc and serum copper levels. Following the initial blood draw, subjects were instructed to take two zinc gluconate lozenges (each containing 13.3 mg of zinc) between breakfast and lunch and two lozenges between dinner and bedtime, for a total of 53.2 mg of zinc per day. They were to avoid taking the lozenges with meals or with any citric acid-containing foods or drinks, nuts, bran, wheat, and oats (sources of phytates) for two hours before and after ingestion of lozenges, in order to avoid problems with zinc absorption. This protocol was followed for a total of fourteen days. Subjects were instructed to notify the investigators if they missed a dose or had any adverse effects. On the morning of the fifteenth day, a repeat fasting blood draw was performed in the same manner. Zinc and plasma copper levels were measured again to determine if the supplementation dose for the allotted time led to a change in plasma zinc levels (along with any observed effect on serum copper). Five of the subjects were instructed to take their final two lozenges within two hours of the second blood draw, while the other ten ingested their final dose the evening prior to blood draws.

The second portion of the study followed the same protocol, except it separated out control subjects and individuals on chronic PPI medications. Five healthy controls and five subjects on chronic PPI medications who met the inclusion criteria were enrolled and underwent the same protocol as above. They had an initial blood draw followed by 53.2 mg of zinc supplementation per day for 14 days. In this arm of the study, all of the subjects took their last dose of zinc lozenges the morning of the second blood draw, on day fifteen.

The third portion of study required only one blood draw and neither group received supplemental zinc. Ten healthy controls and ten patients on chronic PPIs who met the inclusion criteria had 2 vials (10 mL) of venous blood drawn. One vial was sent to Quest Diagnostics for plasma zinc measurements. From the other vial, 1 mL of blood was mixed with 4 volumes of double distilled water (for osmotic shock) and then vortexed at high speed for 30 seconds. The sample was left to sit for 10 minutes at room temperature and then vortexed again at high speed for 10 seconds. Lysed red blood cells (RBCs) were then centrifuged at 10,000 g at 4 °C for 20 minutes. The supernatant was diluted with additional double distilled water and analyzed by atomic absorption spectrophotometry.

Atomic absorption measurements were performed using the PerkinElmer Analyst 800 atomic absorption spectrophotometer equipped with WinLab 32 intuitive software. It utilizes a double beam optical system, solid-state detector, and deuterium background correction. A single slot air-acetylene 10 cm burner head was used for all measurements. Burner height and fuel gas flow were automatically optimized for each element using the WinLab 32 software. Calibration standards in the range of 0.01 to 0.50 ppm were prepared by dilution of a 1000 ppm Zinc AA standard (zinc metal in 3% nitric acid) obtained from Ricca Chemical Company. Standards and samples were diluted with Millipore water (> 18.2 mega ohms) which also served as the blank. Aspirated standards and samples were measured in triplicate.

An additional sample of supernatant was used for a Lowry-based protein analysis (Biorad DC Protein Assay). Results are expressed as micrograms of zinc per milligram of protein.

### Statistics

A paired Student’s t test was used as the criterion for statistical significance (P < 0.05) in all studies described herein.

## Results

A total of 46 subjects enrolled across all 3 studies. All patients except one completed the study and no major adverse events were noted. The patient demographics are illustrated in Table 1.

**Table 1:** Demographic Data

	Study 1 (n = 15)		Study 2 (n = 5)				Study 3 (n = 10)			
	Controls		Controls		PPI users		Controls		PPI Users	
	n	%	n	%	n	%	n	%	n	%
Age (years)										
18 – 30	5	33.3	3	60	3	60	6	60	2	20
31 - 40	2	13.3	1	20	1	20	2	20	1	10
41 – 50	4	26.7	0	0	1	20	0	0	4	40
51 – 60	2	13.3	1	20	0	0	2	20	3	30
> 60	2	13.3	0	0	0	0	0	0	0	0
Gender										
Male	5	33.3	1	20	1	20	5	50	4	40
Female	10	66.7	4	80	4	80	5	50	6	60
Ethnicity										
Caucasian	13	86.7	4	80	5	100	7	70	9	90
Asian	2	13.3	1	20	0	0	3	30	0	0
African American	0	0	0	0	0	0	0	0	1	10
Type of PPI										
n/a										
Protonix					0	0			3	30
Prilosec					2	40			4	40
Nexium					1	20			1	10
Aciphex					1	20			2	20
Prevacid					1	20			0	0
PPI Duration										
n/a										
6 months-2 years									4	40
> 2 years									6	60
GI Disease										
GERD	3	20			5	100	0	0	9	90
Other	0	0			0	0	0	0	1*	10
None	12	80			0	0	10	100		0

* Barretts, Hemochromatosis.

**Figure 1:**
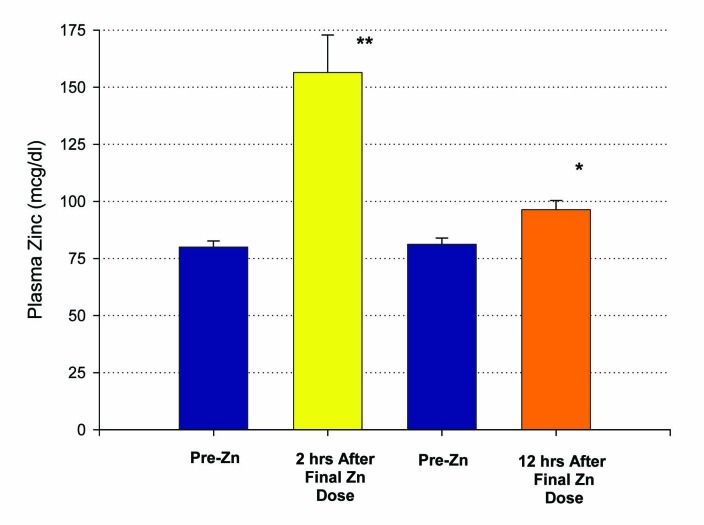
Elevation of plasma zinc after 14 days of 50 mg supplemental Zn per day. Results shown represent the mean ± standard error of the mean. A group of n = 10 healthy control, test subjects had a blood sample taken 12 hours after their final Zn dose. A second group (n = 5), also healthy controls, had their blood sample taken only 2 hours after their final Zn dose. ^*^P < 0.005 (paired Student’s t test); ^**^P < 0.01 against their own pre-zinc supplement plasma Zn values.

**Figure 2:**
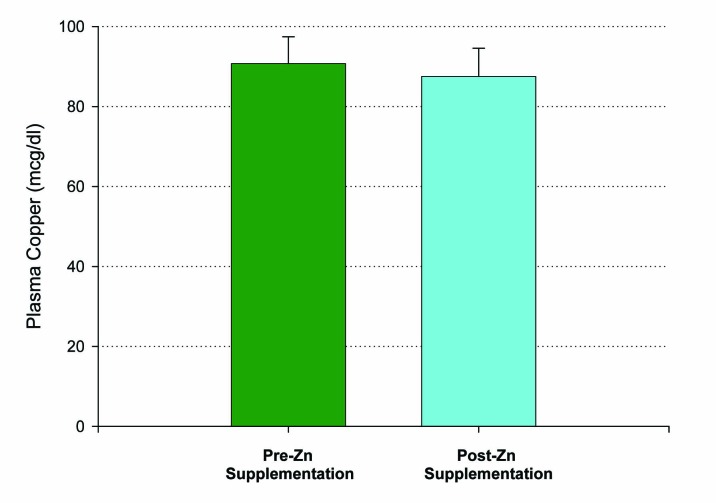
Lack of effect of Zn supplementation on plasma copper levels. A group of n = 10 healthy control, test subjects had their blood sample taken 12 hours after their final Zn dose. P = 0.35 (paired Student’s t). Not significant. Data shown represent mean ± standard error of the mean.

Fifteen patients who met inclusion criteria, from 19 to 63 years of age, were enrolled in this first study. An initial blood sample was taken to evaluate for the baseline levels of plasma zinc. Following a 14 day regimen of 53.2 mg of zinc supplementation a day, the first ten patients had their second blood draw performed 12 hours after the last dose of zinc was ingested. A statistically significant increase in plasma zinc levels was seen from 81 ± 3 mcg/dL to 96 ± 4 mcg/dL (P < 0.005) (Fig. 1). There was very little effect on plasma copper levels with the increased ingestion of zinc over the two week period. A small decrease in copper levels from 91 ± 7 mcg/dL to 88 ± 7 mcg/dL was observed and failed to be significant, with a P = 0.35 (Fig. 2). The remaining five patients took their last zinc dosage 2 hours prior to the second blood sample. These results were again statistically significant, displaying an even higher increase in zinc plasma levels following the 14 day supplementation period from 80 ± 3 mcg/dL to 156 ± 17 mcg/dL with a P < 0.01 (Fig. 1). Again, little effect was seen on plasma copper levels with pre-supplementation values of 136 ± 29 mcg/dL and post-supplementation levels of 128 ± 24 mcg/dL.

Individuals on PPIs were not excluded from the initial set of patients studied. Upon further review of the data collected, subjects on PPIs were noted to have dramatically lower levels of zinc following supplementation compared to those not on PPIs. This prompted our second study, analyzing the efficacy of zinc supplementation in patients on PPI medications.

**Figure 3:**
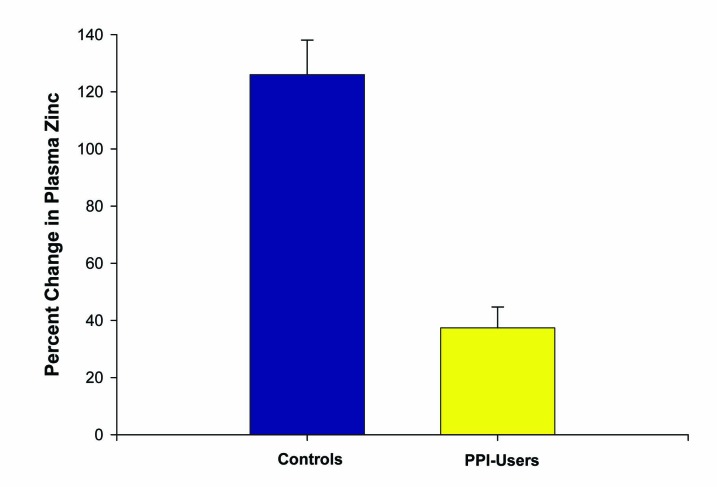
Effect of PPI use on percentage change in plasma zinc levels after zinc supplementation. The increase in plasma zinc levels after 14 days of 53.2 mg/day zinc supplementation was measured for n = 5 healthy control test subjects versus n = 5 reflux-disease patients on long-term PPI therapy. Data shown represent the mean percentage increase ± the standard error of the mean. P < 0.01 (paired Student’s t test).

Six patients on chronic PPIs (at least > 6 months) and five healthy controls, ranging from 17 to 57 years of age, were included in this second study. Again, zinc supplementation was given at 53.2 mg per day over 14 days. This time, all subjects ingested their last zinc dosage 2 hours prior to the second blood draw. One patient in the PPI group dropped out of the study due to GI upset from the lozenges, resulting in a total of ten patients completing this study. Plasma zinc values were increased to a much higher degree among the controls (84 ± 4 mcg/dL to 191 ± 20 mcg/dL) than those on chronic PPI therapy (87 ± 8 mcg/dL to 121 ± 18 mcg/dL). A statistically significant percent increase in plasma zinc of 126 ± 12% was observed in the controls compared to 37 ± 7% in those on chronic PPIs (P < 0.005) (Fig. 3).

**Figure 4:**
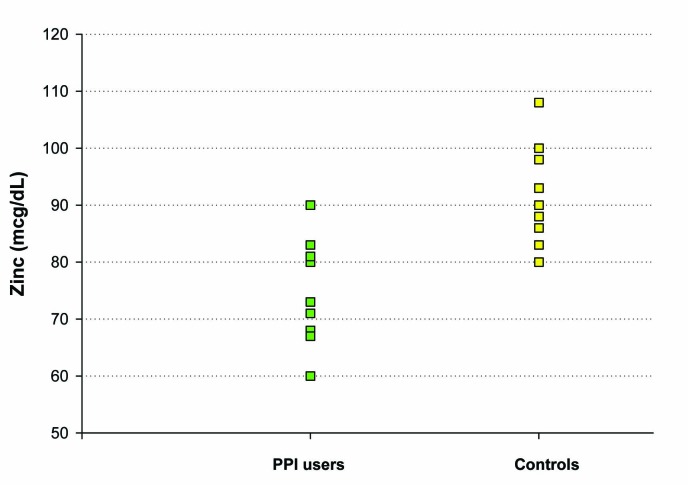
The effect of chronic PPI use on plasma zinc levels. Plasma zinc levels (mcg/dL) were measured in a group of 10 test subjects on long-term PPI medication (as described in Methods) and a matched group of 10 healthy control test subjects not taking PPI, antacid or H2 blocker medication. For the healthy control group, the mean (±standard error of the mean) was 91 mcg/dL ± 3 and the mean for the PPI-group was 75 mcg/dL ± 3 (Paired Student’s t test, P = 0.004).

Our third study evaluated the difference in baseline plasma zinc stores among healthy controls vs. individuals on chronic PPI medications (6 months or longer), without any zinc supplementation given. Ten controls and ten subjects on chronic PPIs, ranging from 17 to 59 years of age, were included in this study. Only a single blood draw was performed in these groups. Both plasma zinc levels and RBC zinc levels were analyzed. A statistically significant difference in baseline plasma zinc stores was seen between the controls (91 ± 3 mcg/dL) and the chronic PPI users (75 ± 3 mcg/dL) (P = 0.004 (Fig. 4). Investigation of RBC zinc between the 2 groups yielded no statistical difference, suggesting a stable pool and adaptable intracellular transport of zinc by RBCs.

## Discussion

PPIs are widely used to treat a variety of gastrointestinal disorders. While no major adverse events have been readily reported with prolonged use, many studies have observed some undesirable effects on the human digestive system. PPIs have been linked to calcium malabsorption, and more recently hypomagnesemia [5, 15]. Deficiencies in both minerals can lead to more serious consequences, including bone fractures and hypokalemia. Zinc is another divalent cation that is pertinent to our homeostasis, and this current study demonstrates that its absorption is also altered with the use of PPIs.

Our results illustrate that individuals on chronic PPIs not only have lower baseline plasma zinc stores, but also an inability to adequately increase zinc plasma levels with oral zinc supplementation. Healthy controls analyzed in the same fashion as test subjects on PPIs had a significantly superior ability to absorb zinc following supplementation. None of the PPI users however were found to be classically zinc-deficient (less than 60 mcg/dL) at baseline [16]. It is believed that marginal deficiencies in zinc intake do not correlate well with plasma zinc values [17]. Also, plasma zinc levels do not vary greatly as a result of mild changes in dietary zinc, requiring more of a drastic and prolonged restriction of zinc intake to manifest a measurable deficiency [18].

Animal studies have shown that cancer incidence can correlate inversely zinc status. Zinc deficiency has shown an impressive increase in chemically-induced esophageal and forestomach tumors in mice. Tumor incidence, following N-nitrosomethylbenzylamine exposure, has been found to range from 57% to 100% in zinc-deficient mice, compared to 17% to 67% in mice who are zinc-sufficient. This was observed with only a 26% difference in serum zinc [19]. Conversely, zinc supplementation in rats with oral cancer, eliciting as little as a 13% increase in plasma zinc, has shown a statistically significant decrease in tumor size, multiplicity, dysplasia, and invasiveness [20]. These studies illustrate the great impact that only mild changes in zinc can impose on certain disease processes.

The subjects in this current study on chronic PPIs were found to have lower baseline plasma zinc levels with a mean of 75 ± 3 mcg/dL compared to 91 ± 3 mcg/dL in healthy controls. This computes to an 18% decrease in baseline zinc stores in chronic PPI users. With the animal data available, this raises the question of what the potential impact of this decrease in plasma zinc may have on the human population.

Even mild zinc deficiency has been linked to a number of chronic, mainly inflammatory, diseases. Lower levels of zinc have been observed in patients with cirrhosis, inflammatory bowel disease, chronic pancreatitis, and infectious diseases [21].

The findings from this study could possibly be extrapolated to a warning concerning the use of PPIs in the pediatric population. PPIs are now often empirically given to infants with nonspecific symptoms; such as recurrent vomiting, anorexia, irritability, excessive crying/colic and chronic cough [22, 23]. There are currently no evidence-based guidelines for the treatment of these disorders in the pediatric population, and little is known about the long-term safety and efficacy of PPI use in these patients. In patients 1 to 17 years old, PPIs have become the first line of treatment for GERD and erosive esophagitis, and a frequent treatment for colic [24].

Infants may be at a greater risk for zinc deficiency (and in greater danger during zinc deficiency) due to increased requirements for and utilization of zinc in growth and development [25]. This increased vulnerability to deficiency continues throughout childhood, and peaks in puberty. Following this time of maximum zinc utility, additional zinc is then required to replete the body’s stores [26]. After observing the potential effects that PPIs have on zinc stores in adults, and their subsequent inappropriate response to supplementation, the detrimental effects that these medications could have on growth and maturity in infants and children may be of concern. A future study focusing on the impact that PPIs have on zinc uptake in the pediatric population is clearly indicated. More valuable still would be the determination of a possible effect of PPI use on the zinc body stores of infants and adolescents.

PPIs are very popular and effective drugs in the general population. As their use and availability have increased, more knowledge of potential adverse effects of these medications has arisen. Our findings of zinc deficiency and impaired zinc absorption during supplementation have added to the previously discovered issues with nutrient and vitamin absorption. Further studies regarding zinc and PPIs are required to expand on these results and thereby help to better ascertain the potential effects of this interaction and subsequent deficiency.

## Supporting Agencies

This work was supported by a grant from the Sharpe-Strumia Research Foundation (JMM).
